# Changes in diet and food shopping behaviors among Asian–American adults due to COVID‐19

**DOI:** 10.1002/osp4.485

**Published:** 2021-02-23

**Authors:** Pasquale E. Rummo, Rhea Naik, Lorna E. Thorpe, Stella S. Yi

**Affiliations:** ^1^ Department of Population Health New York University School of Medicine New York New York USA

**Keywords:** obesity prevention, COVID, diet, food access, food insecurity, health disparities

## Abstract

**Objective:**

COVID‐19 has changed diet and food shopping behaviors, but a lack of disaggregated data by racial and ethnic subgroup makes it challenging to identify whether specific populations are experiencing greater challenges in safely securing an adequate food supply and engaging in healthy eating behaviors during the pandemic. Thus, the objective of this study was to measure such changes among Asian–American (AA) adults, overall and by ethnic subgroup.

**Methods:**

Using a nationally derived nonprobability sample, 3084 AA adults were recruited, including 1737 East Asian, 570 South Asian, 587 Southeast Asian, and 124 multiethnic Asian adults. Participants completed an online survey with questions related to sociodemographics, health status, and diet and food shopping behaviors, including questions related to COVID‐19. Logistic and linear regression were used to compare differences in survey responses by Asian ethnic subgroup.

**Results:**

Compared to other AA subgroups, a higher percentage of Asian Indian (17%), Filipino (13%), Vietnamese (12%), and Korean (11%) adults reported no longer getting food resources they were receiving before COVID‐19 (e.g., mobile meals, food pantry items). The percentage of Filipino (8%) and Vietnamese (7%) adults who reported not having enough money to buy food they need was also higher than other AA subgroups. And a higher percentage of Asian Indian adults (7%) reported not having a way to get to the food store since COVID‐19 than other AA subgroups.

**Conclusions:**

Previous work has not included disaggregated data, which may mask important disparities in food access and food insecurity among people hit hardest by COVID‐19, such as Filipino, Vietnamese, and Asian Indian households.

## 1 INTRODUCTION

There are stark racial and ethnic disparities in the social consequences and health impact of COVID‐19 across the United States, with important differences between racial/ethnic groups. Several reports, for example, indicate an increase in incidents of discrimination among Asian–American (AA) adults, which may contribute to anxieties about leaving home, including trips to the grocery store.^1^ Emerging data from several geographic locations also suggest a higher case fatality rate among AAs compared to other racial/ethnic groups.^2^ The higher case fatality rate may be due to limited healthcare access; lower willingness to be tested; late presentation at the hospital when symptoms are already severe; and increased background prevalence of risk factors for severe illness such as diabetes and hypertension in specific Asian subgroups (e.g., Filipinos, South Asians).^3^ AA adults also make up a large share of essential workers, including healthcare and food retail occupations,^4^, ^5^, ^6^ which may put them at higher risk of infection. While published case rates are low, the true burden of COVID‐19 in the AA population may be underestimated due to race/ethnicity mischaracterization and a lack of data disaggregated by Asian ethnic subgroup.^7^

There is also growing evidence to suggest that COVID‐19 has disrupted food shopping patterns and diet behaviors, potentially due to reduced store hours, food item shortages, and fear of infection.^8^ Results from a nationally representative survey indicate that, although Asian households were not significantly more food insecure than White households in late April, 2020, Asian households were more likely to be afraid to go out to buy food, and were more likely to face transportation issues when purchasing food.^9^ Unfortunately, the researchers were not able to stratify results by Asian ethnic subgroup, which may have masked important heterogeneity in responses among AA adults.

Understanding heterogeneity in changes in food shopping and diet behaviors among AA adults during the COVID‐19 pandemic is an important step towards understanding drivers of recent obesity trends. Recent research from the United Kingdom, for example, suggests that the COVID‐19 crisis (April–May 2020) may have contributed to a decline in weight‐gain protective behaviors (e.g., snacking frequency) and experiencing barriers to weight management,^10^ especially among obese participants and those with a high level of stress.^11^ Although AAs have a historically low prevalence of obesity relative to other racial and ethnic groups,^12^ evidence from local and national studies indicate that obesity has been increasing among AA adults, and is of particular public health concern for specific subgroups such as Filipinos and Asian Indians.^3^ Moreover, anthropomorphic differences (i.e., high percent body fat, low muscle mass) in Asian populations have led to the broad consensus that current definitions of overweight and obesity likely underestimate the true burden of the metabolic effects of obesity in AA.^13^, ^14^, ^15^ Previous work suggests that food insecurity is linked to obesity via irregular eating patterns, including periods of under‐consumption of nutrient‐rich foods when resources are insufficient and overconsumption of low‐cost, calorie‐dense foods when resources are sufficient.^16^ This is concerning because food insecurity is high and participation in the Supplemental Nutrition Assistance Program (SNAP) is low in specific AA subgroups (e.g., Vietnamese households).^17^

The lack of disaggregated data on AA ethnicity makes it challenging to identify whether specific subgroups are experiencing greater challenges in safely securing an adequate food supply and engaging in healthy eating behaviors during the pandemic. Though previous research has reported on changes in weight‐gain related behaviors in the general population,^10^, ^11^ no previous work has examined how COVID‐19 has changed diet and food shopping behaviors of AAs using disaggregated data. Therefore, the objective of this study was to measure such changes among AAs, overall and by ethnic subgroup. It was hypothesized that Southeast Asian adults would be less likely than East Asian adults to have sufficient resources (e.g., money, food assistance benefits) for safely acquiring an adequate supply of food; and South Asian adults would report buying healthier food to prepare for the pandemic.

## 2 METHODS

Using a nationally derived nonprobability sample, 3084 AA adults were recruited from June 9 to 15, 2020. Dynata, an online surveying company that recruits volunteer research participants through their online panels, other online communities, social networks, and Web sites,^18^ was used to recruit the sample. Dynata applies a 3‐stage randomization process to match participants with surveys they are likely to be eligible for and complete, with proprietary quality control procedures to ensure participants do not take the same survey twice. To reduce selection bias associated with the topic of the survey, invitations did not include specific details of the survey. Participants who completed the survey received points that can be redeemed for various incentives, including cash, lotteries, or donations to charity.

Potential participants completed an online consent, followed by a brief pre‐screening questionnaire. Eligibility criteria included identifying as Asian, being age 18 years and older, and English literacy. The survey was completed on either a personal computer, laptop, tablet, or mobile phone. Open REDCap, an online survey platform, was used to create and distribute the survey.^19^ The survey was designed to assess sociodemographics, health status, food shopping behaviors, and changes in behaviors due to COVID‐19. Sociodemographic and food insecurity questions were derived from the 2017‐2018 National Health and Nutrition Examination Survey.^20^ The survey included 10 self‐reported health outcome items from the Patient Reported Outcomes Measurement Information System and a single‐item, self‐reported question of diet quality.^21^, ^22^ A 10‐item version of the Marin Short Acculturation Scale was also included in the survey, which yields a total acculturation score ranging from 10 to 50.^23^ To be consistent with similar research studies, questions regarding changes in food shopping behaviors were derived from a COVID‐19 food survey implemented by nutrition and public health experts at the University of Tennessee, Knoxville.^8^

The sample was recruited to approximately match the distribution of gender and age of Asian adults residing in the U.S.^24^ Duplicate responses (*n* = 37) and implausible skip patterns (*n* = 29) were dropped. The final sample included 1737 East Asian, 570 South Asian, 587 Southeast Asian, and 124 multiethnic Asian (*n* = 3018) adults (Table 1). The median completion time for the survey was 17.4 min (IQR: 12.1, 25.3).

**TABLE 1:** Sample size and Asian–American subgroup classification of study participants

	*N*	%	Subgroup
Chinese/Cantonese	1078	35.7	East
Asian Indian	452	15.0	South
Japanese	419	13.9	East
Filipino	322	10.7	Southeast
Vietnamese	251	8.3	Southeast
Korean	221	7.3	East
Taiwanese	112	3.7	East
Bangladeshi	40	1.3	South
Pakistani	38	1.3	South
Thai	35	1.2	Southeast
Cambodian	32	1.1	Southeast
Laotian	21	0.7	Southeast
East Indian	20	0.7	South
Burmese	19	0.6	South
Bharat	18	0.6	South
Hmong	15	0.5	East
Indonesian	15	0.5	Southeast
Malaysian	15	0.5	Southeast
Okinawan	11	0.4	East
Nepalese	9	0.3	South
Sri Lankan	9	0.3	South
Singaporean	9	0.3	Southeast
Dravidian	6	0.2	South
Bengalese	5	0.2	South
Bhutanese	3	0.1	South
Mong	2	0.1	East
Siamese	2	0.1	Southeast
Iwo Jiman	1	0.0	East
Laohmong	1	0.0	East
Nipponese	1	0.0	East
Goanese	1	0.0	South
Maldivian	1	0.0	South
Indochinese	1	0.0	Southeast
Madagascar/Malagasy	0	0.0	Southeast

### 2.1 Statistical analysis

Descriptive analysis was performed on the total sample, as well as by Asian ethnic subgroup and national identities with a large sample size (Chinese/Cantonese [*n* = 1078], Asian Indian [*n* = 452], Japanese [*n* = 419], Filipino [*n* = 322], Vietnamese [*n* = 251], Korean [*n* = 221], and Taiwanese [*n* = 112]). Logistic and linear regression was used to compare differences in survey responses by Asian ethnic subgroup (East Asian as referent group), applying a two‐sided alpha of 0.05 as the threshold for statistical significance. Stata version 15.1 (StataCorp LP) was used for all analyses.^25^

## 3 RESULTS

In the sample, 82.1% of respondents reported an annual household income above $20,000 and 78.6% of respondents reported having a post‐secondary education degree (Table 2). The average household size was 2.8 persons and 51.7% of respondents reported being married. Approximately 39% of respondents reported being born outside of the U.S., and 60% reported having at least one foreign‐born parent. Compared to East Asian adults (37.0 [SD = 7.8]), a lower acculturation score was observed among Southeast Asian (35.6 [SD = 7.8]) and South Asian (33.2 [SD = 8.1]) adults. In the full sample, 52.9% of respondents reported currently working at a job or business. Compared to East Asian adults, however, Southeast Asian adults were more likely to report a decrease in work hours (9%; *p* = 0.01), and job loss was highest among Vietnamese adults (11%; Table 3).

**TABLE 2:** Sociodemographic characteristics (%), overall and by Asian–American subgroup^a^

	Total	East Asian^b^	South Asian^b^	Southeast Asian^b^	Multi‐ethnic^b^	Asian Indian	Chinese/Cantonese	Filipino	Japanese	Korean	Taiwan ese	Vietnamese
Total (*N*)	3018	1737	570	587	124	883	1078	322	293	251	221	112
Gender												
Male	46.6%	47.2%	49.5%	43.1%	40.3%	48.9%	48.0%	41.0%	48.4%	44.3%	38.4%	42.6%
Female	53.3%	52.6%	50.5%	56.9%	58.1%	51.1%	51.9%	59.0%	51.3%	55.7%	61.6%	57.4%
Other/refused/missing	0.2%	0.2%	0.0%	0.0%	1.6%	0.0%	0.2%	0.0%	0.2%	0.0%	0.0%	0.0%
Age group												
18–24	15.3%	12.2%	19.3%	19.6%	21.0%	18.1%	14.7%	15.2%	6.2%	19.0%	9.8%	30.3%
25–34	21.1%	16.4%	29.8%	25.4%	26.6%	27.4%	18.6%	24.2%	12.2%	18.1%	17.0%	26.7%
35–44	19.5%	17.6%	22.5%	22.5%	18.5%	23.9%	17.0%	24.5%	10.0%	30.8%	26.8%	20.3%
45–54	17.0%	19.2%	11.6%	16.7%	11.3%	11.7%	18.2%	17.1%	19.3%	19.5%	17.9%	11.6%
55–64	12.6%	15.7%	7.9%	8.5%	10.5%	8.4%	14.7%	9.9%	20.8%	8.1%	16.1%	5.6%
65–99	13.8%	18.6%	7.9%	6.5%	7.3%	9.3%	16.7%	8.4%	31.0%	3.6%	12.5%	4.4%
Refused/missing	0.8%	0.3%	1.1%	0.9%	4.8%	1.1%	0.3%	0.6%	0.5%	0.9%	0.0%	1.2%
Country of origin, father												
United States	30.0%	36.1%	20.4%	21.5%	28.2%	19.7%	23.0%	27.6%	71.6%	43.9%	9.8%	15.9%
Outside of the United States	64.4%	60.0%	72.1%	72.6%	52.4%	72.6%	72.8%	67.7%	26.0%	50.2%	87.5%	74.5%
Other/Don't know/Refused/Missing	5.6%	3.9%	7.5%	6.0%	19.4%	7.7%	4.2%	4.7%	2.4%	5.9%	2.7%	9.6%
Country of origin, mother												
United States	26.4%	32.7%	17.7%	15.7%	29.0%	17.5%	21.2%	17.7%	65.9%	36.7%	8.9%	12.7%
Outside of the United States	67.2%	63.6%	72.3%	77.3%	47.6%	72.8%	74.8%	75.5%	32.2%	57.9%	87.5%	77.7%
Other/Don't know/Refused/Missing	6.4%	3.7%	10.0%	7.0%	23.4%	9.7%	4.1%	6.8%	1.9%	5.4%	3.6%	9.6%
Country of origin, at least one parent												
United States	40.2%	44.7%	32.6%	31.5%	53.2%	32.3%	32.6%	34.8%	78.3%	52.9%	18.8%	29.5%
Outside of the United States	59.7%	55.3%	67.4%	68.5%	45.2%	67.7%	67.4%	65.2%	21.7%	47.1%	81.3%	70.5%
Other/don't know/refused/missing	0.1%	0.0%	0.0%	0.0%	1.6%	0.0%	0.0%	0.0%	0.0%	0.0%	0.0%	0.0%
Country of origin, self												
United States	56.3%	63.4%	38.9%	52.0%	56.5%	37.4%	60.9%	46.6%	78.8%	58.4%	42.0%	62.9%
Outside of the United States	39.3%	34.1%	52.8%	44.1%	26.6%	55.1%	36.6%	48.8%	19.3%	38.5%	54.5%	33.5%
Other/don't know/refused/missing	4.5%	2.5%	8.2%	3.9%	16.9%	7.5%	2.5%	4.7%	1.9%	3.2%	3.6%	3.6%
Acculturation score (10–50)	8.0	7.8	8.1	7.8	7.4	8.2	8.0	7.6	6.6	7.2	8.2	7.9
Educational attainment												
Less than 9th grade	0.4%	0.3%	0.2%	0.7%	0.8%	0.2%	0.2%	0.6%	0.2%	0.9%	0.9%	1.6%
9th to 12th grade—No diploma	1.9%	1.5%	1.6%	3.0%	3.6%	1.0%	1.5%	0.9%	0.9%	1.3%	0.9%	5.4%
GED or equivalent	5.8%	4.8%	5.2%	9.7%	3.6%	3.1%	4.8%	10.1%	4.7%	7.5%	4.3%	7.7%
Some college, no degree	12.6%	11.7%	11.9%	15.8%	13.7%	11.9%	12.2%	18.3%	12.6%	11.3%	6.3%	15.5%
Associate's degree	7.5%	7.4%	5.8%	8.7%	11.3%	5.1%	5.9%	9.9%	12.4%	8.6%	2.7%	5.6%
Bachelor's degree	41.3%	42.5%	38.6%	41.7%	35.5%	38.3%	42.4%	42.2%	42.7%	41.6%	38.4%	42.2%
Graduate or Professional degree	29.8%	31.4%	36.0%	19.6%	26.6%	39.8%	32.7%	16.8%	26.3%	28.5%	45.5%	21.1%
Don't know/refused/missing	0.4%	0.3%	0.2%	0.3%	4.0%	0.2%	0.4%	0.6%	0.0%	0.0%	0.9%	0.4%
Household size												
1	18.6%	22.6%	12.5%	13.1%	17.7%	11.1%	21.3%	13.7%	21.7%	28.1%	18.8%	12.4%
2	30.5%	34.1%	24.0%	26.2%	29.8%	26.1%	31.9%	24.8%	44.2%	21.7%	43.8%	23.9%
3	20.6%	19.7%	23.9%	19.6%	21.8%	25.2%	21.3%	21.4%	17.2%	15.4%	19.6%	19.1%
4	20.5%	17.2%	27.4%	24.5%	15.3%	29.2%	18.5%	22.4%	11.9%	24.9%	14.3%	27.1%
5	6.1%	4.4%	5.8%	11.6%	5.6%	4.0%	4.5%	12.1%	3.6%	8.1%	1.8%	13.1%
>5	3.4%	1.8%	6.3%	4.8%	5.6%	4.2%	2.2%	5.6%	1.4%	1.8%	1.8%	4.0%
Don't know/refused/missing	0.4%	0.2%	0.2%	0.2%	4.9%	0.2%	0.3%	0.0%	0.0%	0.0%	0.0%	0.5%
Household income^b^												
Total (median (IQR))	$90,000 ($50,000, $145,000)	$95,000 ($55,000, $150,000)	$90,000 ($50,000, $130,000)	$75,000 ($49,000, $120,000)	$75,750 ($36,000, $145,000)	$95,000 ($56,000, $139,500)	$95,000 ($50,000, $150,000)	$74,500 ($48,000, $120,000)	$97,000 ($60,000, $149,000)	$87,000 ($50,000, $149,999)	$100,000 ($75,000, $160,000)	$75,000 ($50,000, $120,750)
Refused/missing/implausible	21.0%	17.4%	23.2%	25.7%	38.7%	21.9%	18.4%	26.1%	18.1%	15.4%	13.4%	27.9%
Less than $20,000	11.2%	5.0%	8.1%	8.0%	11.3%	7.7%	4.4%	8.7%	5.3%	8.6%	6.3%	5.6%
$20,000 or more	82.1%	89.2%	84.4%	84.7%	69.4%	84.7%	89.3%	83.2%	87.8%	85.5%	87.5%	84.9%
Don't know/refused/missing/implausible	6.7%	5.9%	7.5%	7.3%	19.4%	7.5%	6.3%	8.1%	6.9%	5.9%	6.3%	9.6%
Relationship status												
Married	51.7%	50.6%	59.6%	47.9%	47.6%	62.6%	47.0%	52.8%	57.8%	43.9%	57.1%	36.7%
Widowed	1.4%	1.4%	1.9%	0.7%	0.8%	1.5%	1.9%	1.2%	1.4%	4.5%	3.6%	1.6%
Divorced	4.6%	5.9%	3.2%	2.9%	1.6%	3.8%	4.5%	3.4%	10.0%	0.5%	0.9%	0.8%
Separated	1.1%	1.0%	1.9%	0.7%	0.0%	2.2%	1.2%	0.3%	1.4%	42.5%	32.1%	51.0%
Never married	35.3%	35.3%	29.8%	40.0%	37.1%	27.0%	39.6%	37.0%	24.1%	6.8%	5.4%	7.6%
Living with partner	4.5%	4.3%	2.6%	6.3%	8.1%	2.4%	4.5%	4.7%	4.1%	1.4%	0.9%	2.0%
Don't know/refused/missing	3.0%	2.8%	2.2%	2.9%	9.2%	1.2%	1.3%	1.3%	2.8%	0.8%	0.0%	0.6%
Occupational status												
Working at a job or business	52.9%	54.5%	51.8%	50.8%	46.8%	53.5%	53.4%	46.6%	47.0%	67.0%	59.8%	55.0%
With a job or business but not at work	5.5%	5.5%	5.1%	4.8%	9.7%	4.9%	5.6%	5.9%	6.2%	3.2%	6.3%	4.4%
Looking for work	8.9%	7.0%	12.3%	11.4%	9.7%	11.3%	7.8%	11.8%	7.6%	6.3%	5.4%	9.2%
Not working at a job or business	21.0%	24.8%	15.1%	16.7%	15.3%	14.8%	23.1%	20.2%	34.4%	11.3%	22.3%	12.0%
Part‐time or full‐time student	9.8%	7.3%	12.3%	13.8%	14.5%	12.6%	9.1%	13.0%	4.3%	10.9%	6.3%	17.1%
Don't know/refused/missing	1.9%	0.9%	3.5%	2.6%	4.0%	2.9%	1.0%	2.5%	0.5%	1.4%	0.0%	2.4%
Do you or anyone in your household currently get SNAP?												
Yes	7.7%	5.8%	10.7%	9.7%	12.1%	10.6%	6.0%	11.8%	4.5%	9.5%	4.5%	8.0%
No	87.0%	90.8%	80.7%	83.6%	77.4%	81.9%	90.5%	83.5%	93.6%	84.2%	92.9%	83.3%
Don't know/refused	5.3%	3.4%	8.6%	6.6%	10.5%	7.5%	3.4%	4.7%	1.9%	6.3%	2.7%	8.8%

^a^ The total and Asian ethnic subgroups (East Asian, South Asian, Southeast Asian, Multiethnic Asian) column are inclusive of columns representing responses from national identities [Chinese/Cantonese, Asian Indian, Japanese, Filipino, Vietnamese, Korean, Taiwanese].

^b^ Excludes <$100 and >$1000000.5.

**TABLE 3:** Responses (%) to COVID‐19 questions, overall and by Asian–American subgroup^a^

	Total	East Asian^b^	South Asian^b^	Southeast Asian^b^	Multi‐ethnic^b^	Asian Indian	Chinese/Cantonese	Filipino	Japanese	Korean	Taiwanese	Vietnamese
Total (*N*)	3018	1737	570	587	124	883	1078	322	293	251	221	112
How has your work environment changed since COVID‐19?												
I did not work outside of the home, so no change	26.5%	27.1%	28.9%	23.5%	21.8%	27.0%	25.8%	23.6%	33.2%	18.6%	27.7%	23.1%
I worked outside of the home and still work outside of the home, so no change	13.3%	12.4%	11.6%	**16.7%**	16.9%	11.3%	11.6%	20.2%	11.7%	16.7%	14.3%	14.7%
I now do some of my work inside of the home	11.0%	11.5%	11.6%	9.0%	10.5%	12.2%	10.7%	10.2%	11.5%	17.2%	10.7%	8.4%
I now do all of my work inside of the home	27.4%	28.5%	30.0%	**22.8%**	21.0%	32.7%	31.3%	18.0%	20.3%	29.4%	31.3%	28.3%
My hours outside of the home have been decreased	6.6%	5.6%	6.5%	**8.9%**	**10.5%**	7.3%	5.6%	8.7%	5.0%	7.2%	8.9%	8.0%
My work has been closed or I have lost my job	8.9%	8.8%	7.9%	10.6%	8.9%	6.6%	9.5%	9.0%	8.1%	8.1%	4.5%	11.2%
Other/refused/missing	8.6%	8.4%	4.9%	11.1%	13.4%	3.9%	5.7%	13.4%	15.4%	3.3%	3.7%	8.3%
What did you buy specifically or more of than normal to prepare for COVID‐19?												
Dry cereal	34.6%	31.9%	**46.0%**	33.6%	25.0%	46.5%	32.7%	34.8%	28.6%	33.0%	33.0%	33.5%
Milk or milk alternatives	33.1%	15.8%	**51.6%**	**34.2%**	**37.1%**	50.7%	29.5%	35.1%	19.1%	33.0%	19.6%	33.5%
Other dairy foods	9.6%	4.5%	**15.4%**	9.5%	**13.7%**	16.2%	7.3%	11.2%	7.2%	10.9%	7.1%	9.6%
Beverages	25.8%	14.2%	26.8%	**31.9%**	22.6%	25.2%	24.2%	35.4%	21.7%	29.0%	20.5%	25.9%
Sugar	18.6%	7.4%	**34.2%**	**20.8%**	**23.4%**	34.7%	12.2%	22.0%	13.4%	17.2%	8.9%	21.9%
Flour	27.4%	14.5%	**39.6%**	23.9%	31.5%	40.9%	25.3%	27.0%	22.9%	24.0%	28.6%	23.5%
Fruit	39.7%	21.1%	**50.0%**	**41.6%**	**45.2%**	51.1%	39.1%	43.2%	28.6%	33.5%	36.6%	37.5%
Vegetables	43.2%	24.0%	**52.6%**	42.6%	48.4%	53.5%	44.0%	46.0%	32.5%	38.0%	35.7%	37.1%
Condiments	11.9%	5.4%	**16.8%**	**15.0%**	**16.9%**	16.2%	9.5%	18.3%	7.2%	11.3%	8.0%	8.8%
Fish and/or seafood	22.4%	12.2%	20.0%	**30.8%**	22.6%	19.5%	22.8%	31.7%	14.8%	17.6%	20.5%	23.9%
Oils	19.5%	9.3%	**29.1%**	**21.0%**	**25.0%**	30.3%	16.2%	21.4%	13.4%	17.6%	17.9%	17.9%
Butter or margarine	19.0%	9.7%	**23.7%**	**22.7%**	20.2%	24.1%	15.3%	28.6%	19.8%	19.9%	11.6%	15.1%
Frozen meals	26.9%	15.3%	25.1%	**33.6%**	24.2%	25.4%	26.8%	32.3%	23.6%	24.0%	28.6%	35.9%
Soups	23.5%	14.5%	20.9%	25.6%	17.7%	20.1%	23.2%	29.5%	28.9%	19.5%	25.9%	21.5%
Peanut or other nut butter	19.9%	10.9%	20.0%	**25.0%**	20.2%	21.2%	18.8%	29.8%	18.6%	18.1%	16.1%	21.9%
Nuts or seeds	17.8%	12.0%	**9.3%**	19.1%	19.4%	27.9%	19.7%	20.5%	19.6%	19.5%	23.2%	15.9%
Beans/legumes	16.6%	8.5%	**25.6%**	14.3%	18.5%	27.7%	14.1%	18.6%	16.5%	15.4%	13.4%	10.4%
Salsa	10.0%	4.5%	**14.7%**	**11.8%**	**16.1%**	15.7%	7.2%	14.9%	8.4%	9.0%	7.1%	9.2%
Crackers	21.6%	12.7%	23.2%	21.8%	21.0%	24.6%	22.0%	27.6%	20.3%	22.6%	21.4%	17.1%
Pretzels	8.3%	3.9%	**10.7%**	**9.9%**	15.3%	11.5%	6.3%	13.0%	6.0%	10.0%	8.0%	7.6%
Chips	29.0%	15.7%	**31.8%**	**34.2%**	31.5%	33.6%	27.6%	39.8%	27.9%	25.3%	24.1%	28.7%
Cheese	22.6%	11.3%	**28.8%**	**27.1%**	24.2%	29.4%	20.0%	35.7%	20.0%	17.2%	16.1%	17.1%
Meat	34.3%	21.1%	**23.3%**	**41.1%**	41.1%	22.6%	39.0%	50.0%	29.4%	33.0%	32.1%	31.9%
Tofu or meat‐substitutes	14.8%	9.5%	**12.1%**	13.8%	19.4%	11.1%	16.9%	13.4%	11.5%	16.7%	20.5%	14.7%
Bread	31.0%	16.4%	**38.4%**	**34.2%**	33.9%	38.1%	28.9%	40.7%	24.6%	28.1%	27.7%	29.5%
Rice	41.5%	24.0%	40.9%	**45.5%**	47.6%	40.7%	41.5%	50.0%	38.9%	38.9%	37.5%	42.6%
Chocolate or candy	16.7%	8.5%	**19.6%**	**19.8%**	**24.2%**	20.1%	15.4%	25.2%	13.8%	16.7%	13.4%	14.7%
Cookies or baked sweets	13.8%	6.4%	**18.2%**	**18.4%**	14.5%	18.6%	12.1%	20.5%	9.8%	12.2%	8.0%	16.7%
Breakfast bars or snack bars	12.9%	6.9%	**16.7%**	13.5%	12.9%	18.1%	11.6%	17.4%	13.6%	12.2%	11.6%	8.8%
Oatmeal, grits, or other cereal to cook	16.7%	9.4%	16.3%	**19.8%**	19.4%	17.5%	16.0%	24.5%	14.6%	18.6%	16.1%	14.7%
Eggs, egg whites, or egg substitutes	35.7%	20.9%	32.8%	**41.2%**	35.5%	32.1%	37.8%	45.7%	31.0%	31.7%	33.9%	33.9%
Ice cream or other frozen desserts	18.5%	10.1%	**21.8%**	19.8%	21.0%	23.0%	18.3%	24.2%	14.1%	22.6%	12.5%	17.9%
Popcorn	12.4%	6.1%	**16.0%**	**14.7%**	**16.1%**	17.0%	10.5%	20.8%	11.0%	12.7%	8.0%	8.4%
Pancake or waffles	11.0%	4.9%	**14.4%**	**15.5%**	**13.7%**	13.9%	8.2%	18.6%	8.1%	12.7%	6.3%	13.1%
Jellos, puddings, custards	4.7%	2.1%	**6.1%**	**6.3%**	**8.1%**	6.6%	3.3%	9.0%	4.5%	3.2%	5.4%	3.6%
Pasta, spaghetti, or other noodles	29.4%	18.4%	**22.6%**	31.0%	35.5%	22.6%	31.2%	38.2%	33.2%	32.1%	26.8%	24.3%
Syrup or honey	9.1%	3.9%	**15.6%**	**9.9%**	**12.1%**	17.0%	6.2%	13.4%	5.0%	12.2%	11.6%	6.8%
Other baking supplies	8.6%	4.4%	**10.0%**	9.5%	**15.3%**	10.4%	7.6%	11.2%	8.8%	10.0%	7.1%	10.0%
Other microwave/quick cook foods	12.3%	6.9%	10.7%	**16.2%**	12.9%	11.3%	12.1%	16.1%	11.9%	11.3%	10.7%	16.7%
Other processed and packaged ready to eat shelf‐stable foods	12.9%	8.1%	**8.9%**	14.8%	15.3%	10.6%	13.4%	15.2%	15.5%	16.3%	14.3%	16.7%
Other processed and packaged ready to eat refrigerated foods	9.9%	5.2%	8.4%	**14.1%**	**14.5%**	8.8%	9.7%	14.0%	9.8%	7.2%	9.8%	13.9%
Creamer or non‐dairy creamer	8.5%	4.6%	7.2%	**12.1%**	10.5%	7.5%	7.7%	16.5%	9.3%	10.4%	8.9%	7.6%
Alcohol	10.5%	6.5%	9.5%	10.1%	12.1%	10.6%	8.3%	11.5%	16.7%	15.4%	10.7%	9.6%
Other	3.7%	2.6%	2.6%	2.6%	6.5%	2.7%	4.3%	2.8%	6.2%	3.2%	4.5%	4.0%
Do you have enough money to buy the food you need during COVID‐19?												
I have enough money to buy the food I need	77.2%	84.0%	**69.3%**	**67.5%**	**64.5%**	70.8%	82.9%	68.0%	88.3%	77.4%	86.6%	69.7%
I have enough to buy some of the food I need	17.2%	12.7%	**24.7%**	**22.1%**	**23.4%**	24.3%	13.6%	21.7%	9.1%	16.3%	11.6%	21.1%
I do not have enough money to buy the food I need	4.4%	2.9%	4.4%	**8.0%**	**9.7%**	4.0%	3.1%	8.1%	2.1%	5.9%	0.9%	7.2%
Refused/missing	1.1%	0.5%	1.6%	2.4%	2.4%	0.9%	0.4%	2.2%	0.5%	0.5%	0.9%	2.0%
Compared to before COVID‐19, are you eating less, more, or the same?												
A lot less	3.1%	2.7%	4.2%	2.9%	5.6%	4.6%	3.2%	3.1%	1.7%	2.3%	4.5%	3.6%
Somewhat less	17.9%	16.5%	**23.5%**	14.5%	**27.4%**	22.3%	17.0%	15.5%	16.0%	17.2%	19.6%	15.1%
The same	57.6%	60.6%	**52.3%**	56.4%	**46.0%**	51.5%	61.1%	54.3%	62.3%	51.6%	58.0%	56.6%
Somewhat more	17.2%	17.0%	16.0%	19.4%	16.1%	17.3%	16.0%	20.8%	16.5%	25.3%	15.2%	18.7%
A lot more	3.5%	2.8%	3.5%	**5.8%**	2.4%	3.3%	2.4%	5.0%	3.1%	3.6%	0.9%	5.2%
Refused/missing	0.6%	0.4%	0.5%	1.0%	2.4%	0.9%	0.4%	1.2%	0.5%	0.0%	1.8%	0.8%
Compared to before COVID‐19, how healthy is your overall diet?												
A lot less healthy	3.7%	2.7%	**5.4%**	4.3%	**8.1%**	6.0%	2.7%	3.7%	3.1%	3.2%	0.9%	6.0%
Somewhat less healthy	20.3%	20.0%	19.3%	22.7%	19.4%	18.8%	18.1%	22.7%	19.1%	25.8%	24.1%	25.1%
The same	53.2%	57.5%	**45.8%**	**48.4%**	50.0%	45.1%	57.9%	49.7%	59.7%	51.1%	56.3%	44.2%
Somewhat more healthy	16.8%	15.2%	**21.2%**	17.2%	16.1%	22.3%	17.1%	15.8%	13.6%	13.1%	15.2%	18.3%
A lot more healthy	5.5%	4.3%	**7.9%**	**7.0%**	4.0%	7.5%	3.9%	7.5%	4.1%	6.8%	3.6%	5.2%
Refused/missing	0.5%	0.3%	0.4%	0.5%	2.4%	0.2%	0.4%	0.6%	0.5%	0.0%	0.0%	1.2%
Do you have a way to get to the store for food since COVID‐19												
Yes	84.3%	89.5%	**70.7%**	**83.3%**	**78.2%**	69.5%	88.7%	85.4%	95.0%	86.9%	81.3%	81.7%
Sometimes	9.8%	6.0%	**18.6%**	**10.9%**	**17.7%**	20.8%	6.4%	10.6%	3.1%	8.1%	8.9%	10.8%
No	3.6%	2.6%	**7.4%**	3.6%	0.8%	7.1%	2.5%	2.8%	1.4%	3.6%	4.5%	4.8%
I would not be going to the store to shop for food anyway, so this does not apply to me	1.9%	1.6%	**3.0%**	2.0%	1.6%	2.7%	1.9%	1.2%	0.5%	1.4%	5.4%	2.4%
Refused	0.4%	0.3%	0.4%	0.2%	1.6%	0.0%	0.6%	0.0%	0.0%	0.0%	0.0%	0.4%
Have you gone to the store for food in the last week?												
Yes	85.9%	87.9%	**78.1%**	87.9%	84.7%	78.8%	87.5%	87.0%	90.2%	89.1%	77.7%	87.3%
No	13.4%	11.6%	**20.4%**	11.8%	13.7%	20.4%	11.9%	13.0%	9.5%	10.4%	22.3%	12.4%
Refused	0.7%	0.5%	1.6%	0.3%	1.6%	0.9%	0.6%	0.0%	0.2%	0.5%	0.0%	0.4%
In the past week, were you able to get the food you were looking for when you were at the store?												
Yes, the store had everything I needed	50.3%	50.8%	48.1%	51.4%	46.8%	49.8%	50.4%	48.8%	54.4%	47.1%	44.6%	53.0%
The store had most of what I needed	31.8%	34.4%	**24.4%**	32.0%	29.8%	24.1%	34.4%	33.5%	33.2%	37.1%	31.3%	29.9%
The store had only a little of what I needed	2.9%	2.0%	**4.6%**	**3.4%**	**6.5%**	4.2%	1.7%	4.3%	2.1%	4.1%	1.8%	2.4%
The store had nothing that I needed	0.2%	0.2%	0.4%	0.2%	0.0%	0.2%	0.3%	0.3%	0.2%	0.9%	22.3%	0.4%
Refused/missing	14.7%	12.6%	22.6%	12.9%	16.9%	21.7%	13.3%	13.0%	10.0%	10.9%	0.0%	14.3%
Are there other ways you are getting food at home that you were not using before COVID‐19?												
None	35.3%	39.5%	31.2%	33.8%	21.1%	32.3%	4.9%	26.2%	39.0%	29.2%	40.0%	53.1%
Online orders	39.3%	38.6%	39.2%	42.3%	36.8%	41.7%	4.9%	42.9%	39.0%	50.0%	32.0%	28.1%
Neighbors	11.5%	9.0%	16.0%	11.3%	10.5%	13.5%	1.3%	14.3%	9.8%	4.2%	8.0%	9.4%
Family	17.9%	12.9%	20.8%	22.5%	**36.8%**	21.9%	1.9%	21.4%	12.2%	8.3%	16.0%	25.0%
Food delivery business	17.9%	18.6%	12.0%	25.4%	21.1%	12.5%	2.6%	26.2%	17.1%	25.0%	8.0%	25.0%
Food from my child's school to eat at home	5.4%	4.8%	4.8%	5.6%	15.8%	4.2%	0.6%	9.5%	4.9%	4.2%	4.0%	6.3%
Other/refused	6.4%	5.2%	6.4%	7.0%	15.8%	5.2%	0.5%	9.5%	4.9%	8.3%	16.0%	0.0%
Are there food resources you were receiving before COVID‐19 that you are now not getting (mobile meals, food pantry, etc.)?												
There are no other food resources I was receiving	68.7%	74.1%	**60.0%**	**63.0%**	**60.5%**	61.1%	71.2%	61.8%	79.5%	71.5%	76.8%	64.5%
There are resources I am no longer receiving	10.8%	7.8%	**17.2%**	**12.6%**	**15.3%**	16.8%	8.3%	13.0%	5.7%	11.3%	5.4%	12.0%
I am still receiving all of the resources I was getting before	16.2%	14.7%	17.7%	**18.2%**	20.2%	18.6%	16.8%	19.6%	11.5%	14.0%	14.3%	15.5%
Refused/missing	4.2%	3.3%	5.1%	6.1%	4.0%	3.5%	3.6%	5.6%	3.3%	3.2%	3.6%	8.0%
Why are you not getting those resources?												
Lack of transportation	19.3%	8.4%	**8.1%**	2.6%	1.3%	6.2%	1.8%	1.3%	1.0%	1.9%	0.3%	1.6%
Reduced hours of service	38.2%	13.3%	**16.2%**	**9.1%**	1.9%	12.7%	2.7%	4.9%	1.6%	3.2%	0.3%	2.6%
Closed or not offered anymore	35.5%	14.9%	**10.7%**	**10.1%**	1.9%	8.1%	3.2%	5.8%	2.6%	1.9%	0.3%	3.9%
I am worried about others having COVID‐19	40.7%	19.8%	**10.7%**	10.1%	2.6%	8.1%	3.5%	4.9%	3.6%	3.9%	1.3%	3.9%
Other/refused	4.0%	1.9%	0.3%	1.0%	1.0%	1.6%	0.5%	1.9%	1.9%	0.6%	0.0%	0.6%

*Note:* Bold denotes statistical significance using a two‐sided alpha of 0.05.

^a^ The total and Asian ethnic subgroups (East Asian, South Asian, Southeast Asian, Multiethnic Asian) column are inclusive of columns representing responses from national identities [Chinese, Asian Indian, Japanese, Filipino, Cantonese, Vietnamese, Korean, Taiwanese].

^b^ Logistic and linear regression were used to compare differences in survey responses by Asian ethnic subgroup (East Asian as referent group).

Approximately 11% of respondents reported there were food resources they were receiving before COVID‐19 that they are now not getting, with a higher percentage among Korean, Asian Indian, Filipino, and Vietnamese adults (Table 3). Participation in SNAP was also higher among these subgroups; and Filipino and Vietnamese adults were more likely to report not having enough money to buy the food they need. Compared to East Asian adults, a higher percentage of South Asian adults reported not having a way to get to the food store since COVID‐19 than other groups (*p* < 0.001), including Asian Indian adults; and a higher percentage of South Asian adults reported not going to the food store in the last week (*p* < 0.001). In addition, a higher percentage of Korean, Asian Indian, and Filipino adults reported that the store only had a little of what they needed.

A lower percentage of East Asian adults reported buying more fruits, vegetables, fish/seafood, and beans/legumes to prepare for COVID‐19 (Table 3). Whereas, a higher percentage of Asian Indian adults reported buying more fruits, vegetables, and bean/legumes to prepare for COVID‐19. A higher percentage of Asian Indian adults also reported eating a lot less or somewhat less compared to before COVID‐19. In contrast, a higher percentage of Filipino adults reported buying more meat, fish/seafood, noodles, and rice; and a higher percentage of Filipino adults and Vietnamese adults reported eating a lot more.

## 4 DISCUSSION

In this study, South and Southeast Asian adults had disproportionate problems with getting adequate food resources as a result of COVID‐19 compared to East Asian adults. For example, there were more salient economic challenges reported by Filipino and Vietnamese adults, including changes in work hours, job loss, and insufficient funds for food shopping. This is concerning because previous work suggests that food insecurity is associated with lower diet quality among non‐Hispanic Asian adults.^26^ In fact, more Filipino and Vietnamese adults reported buying meat and starchy foods (e.g., rice, noodles) to prepare for COVID‐19. Uniquely, Asian Indian adults, the least acculturated group in the sample, most frequently reported experiencing obstacles related to food access, including ways of getting to the physical store and the store not having everything they needed. However, Asian Indian adults also reported a number of healthier changes in diet habits, including buying more fruits, vegetables, and beans/legumes to prepare for COVID‐19. To reduce food insecurity and bolster efforts to improve food accessibility among high‐risk Asian adults, policymakers and public institutions should assist community members in enrolling in food assistance programs and provide additional income relief, and mitigate potential issues related to food access (e.g., increasing the capacity of local food supply chains).

Compared to respondents in a similar survey implemented by the University of Tennessee, in which AA comprised 7% of the sample, a higher percentage of respondents in the survey reported that stores had everything they needed (50% vs. 13%).^8^ Yet, more respondents in the survey reported that there are food resources they are no longer receiving (11% vs. 5%) and not having a way to get to the food store since COVID‐19 (7% vs. 1%). These differences provide some evidence that AAs may have experienced more challenges related to food access relative to the larger U.S. population during an acute phase of the pandemic. This may potentially explain why 65 percent of respondents in the survey reported getting food at home using other ways, such as online orders and food delivery businesses. Whereas, only 32% of respondents reported getting food in new ways in the sample with aggregated data.^8^

This study had a few limitations. The respondents, for example, had higher levels of income and education than the total AA population in the U.S., and the survey was not offered in other languages. The participants' geographic region was also not ascertained. Though the survey was anonymous, it is also possible that social desirability may have affected participants' responses. That said, a key strength of the study was the focus on collecting disaggregated data, which enabled us to identify differences by Asian ethnic subgroups. In contrast, previous surveys, including industry data,^27^, ^28^ have not collected or reported disaggregated data, which may mask important disparities in food access and food insecurity, and thus key differences in obesity risk, among groups hit hardest by the COVID‐19 pandemic, such as Filipino, Vietnamese, and Asian Indian communities. Thus, future research should collect and report survey responses by racial and ethnic subgroups, especially Asian ethnic subgroups, which often are lumped together or classified as “other” or “multi‐ethnic.”

## AUTHOR CONTRIBUTIONS

Pasquale E. Rummo, Lorna E. Thorpe, and Stella Yi conceived of the study design. Pasquale E. Rummo analyzed and interpreted the data. Rhea Naik was involved in the literature search and generating tables. All authors were involved in writing the paper and had final approval of the submitted and published versions.
